# Nerves of the Heart

**Published:** 1858-06

**Authors:** 


*Nerves of the Heart.*—Dr. Robert Lee, of London, who long ago acquired a high reputation by the demonstration of the nerves of the uterus, has recently followed in a still more brilliant discovery. He has succeeded in demonstrating a great quantity of nervous filaments and ganglia presiding over the movements of the heart. He has shown these to be present at all periods of life, and in all the mammalia he has yet examined, and it increases in size in certain diseased conditions in which the muscular substance is hypertrophied, and the motions of the heart strengthened. The paper, which is published in the Medical Times and Gazette for September 12th, 1857, is illustrated by two wood cuts, one of the heart of a child, the other of a portion of that of a heifer. From these it would appear that the cardiac or aortic plexus, long since described by Fallopius, gives off numerous branches, which ramify in all directions over the surface of the heart, swelling here and there in ganglionic enlargements, especially where they cross blood-vessels. These nerves are all soft, flat and somewhat semi-transparent, of a gray color, and invested as to their smaller branches with a neuri-lèmma.

The author states that the chief difficulty in the demonstration of these nerves, arise not from their softness nor the amount of fat in which they are imbedded, but from the presence of a dense fibrous tissue closely investing the heart and sending out prolongations along the vessels and nerves, as well as the fasiculi of muscular fibre. This tissue, which he describes as lying directly under the pericardium, he calls the cardiac fascia.—*American Journal of Dental Science.*

